# Ultrafast electron holes in plasma phase space dynamics

**DOI:** 10.1038/s41598-021-95652-w

**Published:** 2021-08-11

**Authors:** Seyyed Mehdi Hosseini Jenab, Gert Brodin, James Juno, Ioannis Kourakis

**Affiliations:** 1 Department of Physics, Chalmers University of Technology, 412 96 Göteborg, Sweden; 2 Department of Electrical, Computer and Biomedical Engineering, Ryerson University, Toronto, M5B 2K3 Canada; 3 Department of Physics, Umeå University, Umeå, Sweden; 4 Department of Physics and Astronomy, University of Iowa, Iowa City, IA 52242 USA; 5 Department of Mathematics, Khalifa University of Science and Technology, Abu Dhabi, UAE

**Keywords:** Plasma physics, Computational science

## Abstract

Electron holes (EH) are localized modes in plasma kinetic theory which appear as vortices in phase space. Earlier research on EH is based on the Schamel distribution function (df). A novel df is proposed here, generalizing the original Schamel df in a recursive manner. Nonlinear solutions obtained by kinetic simulations are presented, with velocities twice the electron thermal speed. Using 1D-1V kinetic simulations, their propagation characteristics are traced and their stability is established by studying their long-time evolution and their behavior through mutual collisions.

## Introduction

Plasma phase-space dynamics is tacitly characterized by the occurrence of electron holes, a term describing a localized plasma region where electrons are trapped by the electric potential stemming from their own self-generated density variation, as a localized electron depletion region occurs in a self-consistent manner. An electron hole is thus manifested as a localized “trapped” electron population traveling alongside an electrostatic potential disturbance^1,2^. Electron-holes present two main characteristics^3^: a localized positive potential structure which traps electrons, and a symmetry in the electric potential profile around the peak. In addition, electron holes are a type of Bernstein, Greene, and Kruskal (BGK) mode^4^. Electron holes have been observed and studied in laboratory experiments^5^, in space measurements^6–10^ and in kinetic simulations^11^.

In order to construct electron holes in a self-consistent manner within a kinetic model, one may either start with an arbitrary potential profile and then proceed by deriving the distribution function (df) of an electron hole, or, inversely, start with a predefined df for the trapped electrons and thus derive the associated potential profile. The former (integral equation) method, due to Bernstein, Greene and Kruskal^4^ leads to an infinity of solutions whose dynamical stability is not prescribed. The latter (differential equation) method, suggested by Schamel^12–15^, is based on a parametrized *df* (henceforth referred to as “the Schamel df”) allowing one to prescribe the shape of the trapped population (i.e. by assigning a value to parameter $\beta$ associated with the inverse temperature of the trapped population). Recently, Schamel df is extended by adding new parameters and hence resulted in variety of new solutions. Note, most of the solutions, i.e. $\phi (x)$ are undisclosed^16^. In the case of double layers, the Schamel df provides solutions which are much faster than the thermal velocity^17^. In fact, as the authors in Ref.^17^ have predicted, a strong double layer (DL) solution is obtained as a limiting variant of a solitary hole; see also^1^ for details. The Schamel method combined with the pseudopotential approach^18^ may provide initial conditions for a controlled numerical investigation of EH dynamics^19^. Recent studies^19,20^ have shown that the Schamel-pseudopotential approach can produce nonlinear solutions with Mach numbers $1.0< M < 10.0$.

However, only solutions in the range $1.0< M < 3.0$ are found to be stable for long times^19^ and to survive mutual collisions^20^. In other words, structures are destabilized as the Mach number increases. This has been suggested in other kinetic simulations^21^. For very high Mach number ($M > 10$), the Schamel-pseudopotential method can not provide any solutions even for a wide range of $\beta$ (values)^3,19^. The maximum speed for a soliton accompanied by an electron hole (SEH) is $M=1.307$ when using the pseudopotential appoach in the small-amplitude regime^22^.

Despite these theoretical challenges, the existence of high-speed electron holes is a topic of intense study, first getting attention due to observations by the FAST satellite^6,23,24^. Saeki et al.^5^ studied electron holes experimentally using a Q-plasma machine and also via kinetic simulations; they reported structures moving at the electron thermal speed, which they identified as solitons. Solitons are nonlinear structures that can survive mutual collisions and are characterized by a phase shift during a collision^25–29^. We note however, Saeki et al. did not consider the phase shift separating the hole trajectories before and after collisions. It is interesting to point out that fast (large Mach number) localized structures have also been predicted recently, in the form of supersolitons (supernonlinear waves); see e.g.^30–32^. Nonetheless, it is important to realize that these structures are distinct in both their structural characteristics (shape) and in the physical mechanism underlying their formation. (An interested reader is referred to the above references for details).

**Figure 1:**
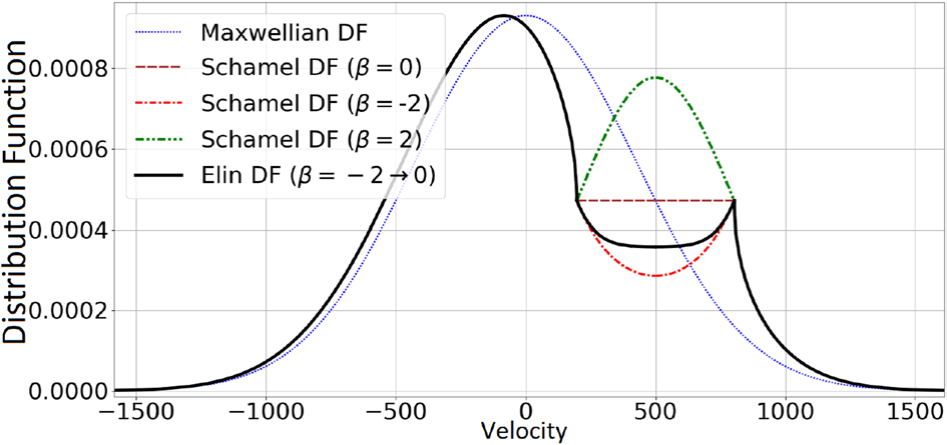
Different distribution functions for the trapped electron population are presented. The Maxwellian df (in the absence of trapped particles) is shown for sake of comparison (blue, thin dotted line). Three shapes of the Schamel df are displayed, namely flat (brown dashed, $\beta =0$), hollow (red, dashed-dotted, $\beta =-2$) and a bump (green, dashed-dotted, $\beta =2$), for $\phi = 25$. The ELIN df (black thick line) is shown when ten carving ($\phi _1 = 2.5$, $\phi _2=5$, $\phi _3=7.5$, ..., $\phi _{10} = 25$) is carried out with their corresponding $\beta$ ($\beta _1 = -2, \beta _2=-1.8, \beta _3=-1.6, \ldots , \beta _{10}=0$).

The aim of this study is to characterize high-speed electron holes by establishing their occurrence in a kinetic framework, and by investigating their stability profile and probing their soliton-like features. For this purpose, a novel distribution function (*df*), the ‘ELIN df’, is introduced as a generalization of the Schamel df. The ELIN df adjusts the distribution function of the trapped population of electrons by relying on a dynamically varying parameter $\beta$ so that its moments can fit a predetermined curve and all of the desired featured of the Schamel df are retained, such as consistency and smoothness in both spatial and velocity spaces inside the trapped region (Fig. 1).

To show the stability of our nonlinear solutions, three series of simulations are reported. Firstly, by considering the long-time evolution of an initial condition we will confirm the stability of the solution’s profile during propagation, thus establishing them as solitary waves. Then, two types of mutual collisions are reported, i.e. head-on collisions (with no overlapping in velocity space) and overtaking collisions (moving in parallel and with overlapping). The aforementioned phase shift through collisions has also been investigated, to corroborate the fact that electron holes behave as solitons.

## Results

### Long-term evolution

**Figure 2:**
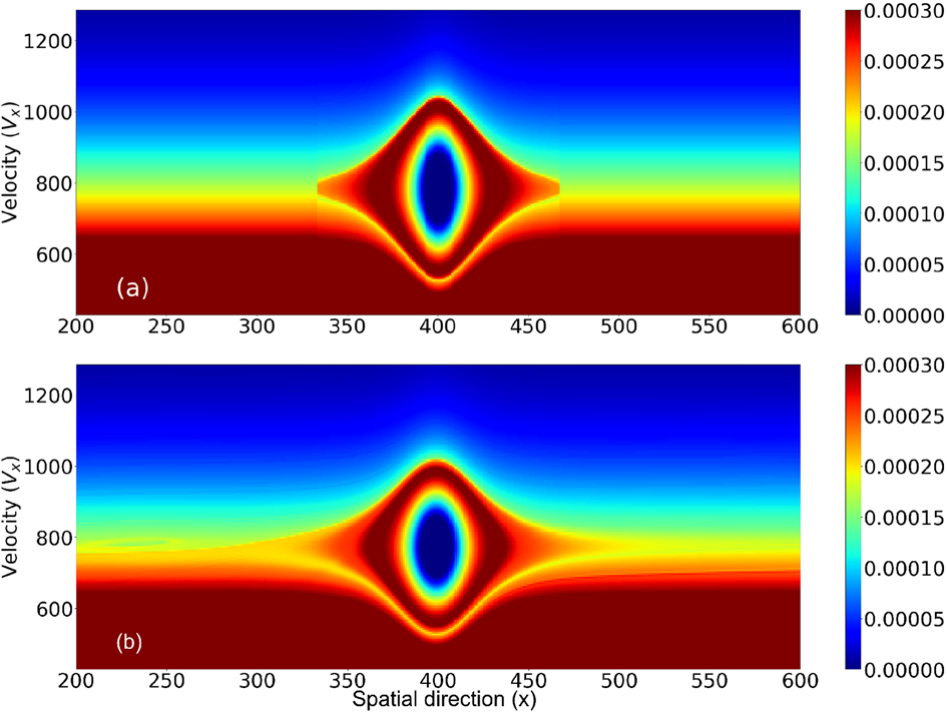
The electron phase space is shown for the case *EH*1 (**a**) at the initial step and (**b**) at $\tau = 12$.

**Figure 3:**
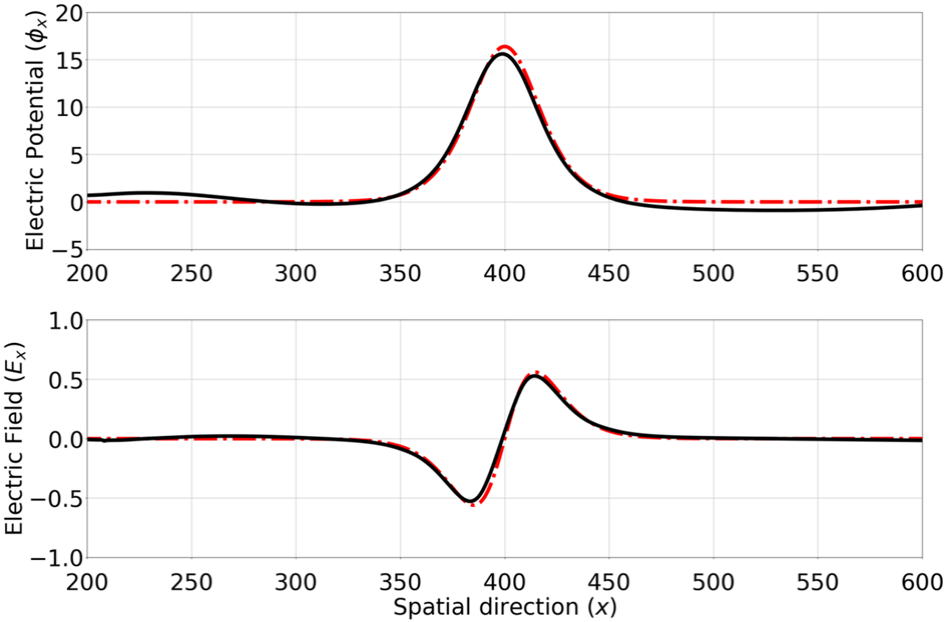
The electrostatic potential/E-field profile of *EH*1 is shown in the top/bottom panel. The initial condition i.e. at $\tau =0$ (red dotted curve) is compared with $\tau =12.0$ (solid black curve), showing a good agreement and hence confirming the stability of EHs during long-time propagation.

Figures 2 and 3 display the temporal evolution of *EH*1. The initial condition and the last step of temporal evolution can be compared and show that the overall shape of the electron hole (Fig. 2) and the corresponding potential or field profile (Fig. 3)stay unperturbed.

### Head-on collision

**Figure 4:**
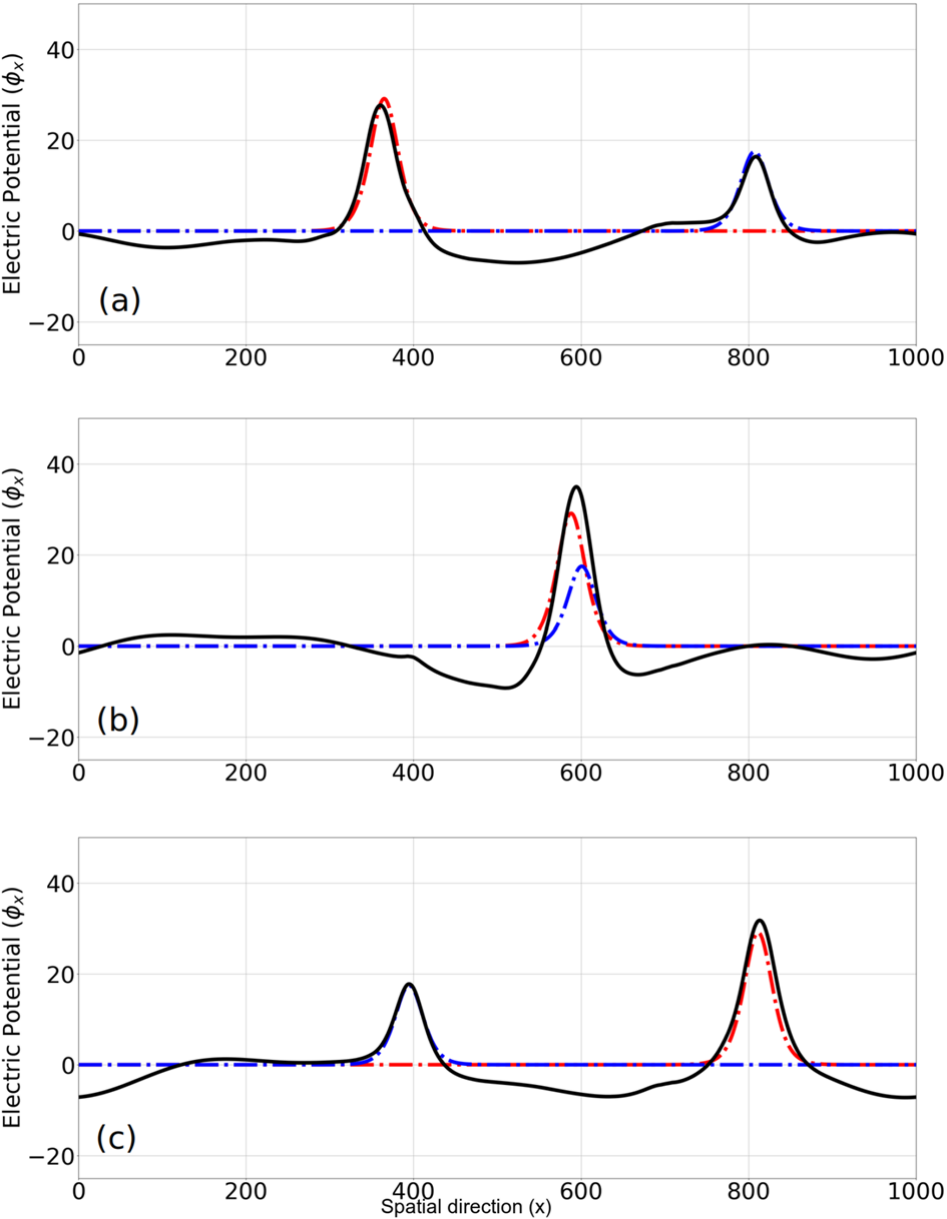
The electrostatic potential profile of *EH*1 and *EH*2 is shown at different snapshots around a head-on collision, namely (**a**) before ($\tau =1.3$), (**b**) during ($\tau =1.6$) and (**c**) after ($\tau =1.9$) the collision. Dotted lines represent the initial condition for each of the solitary wave as if they are propagating without any numerical noise or collisions. Red/blue is for EH1/EH2 which is propagating to the right/left. After the collision, the overall shape and velocity of the solitary wave remains intact.

Figure 4 depicts a head-on collision between *EH*1 and *EH*2. After the collision ($0< \tau < 2$), both solutions keep their shape and velocity compared to their initial state. Note that due to their large velocity, they are well-apart in the velocity direction, i.e. there is no overlapping, and hence their collision on the phase space consists of two electron holes passing each other without much interaction. Both electron holes follow their unperturbed trajectories after the collision, hence no phase shift is observed.

### Overtaking collision

**Figure 5:**
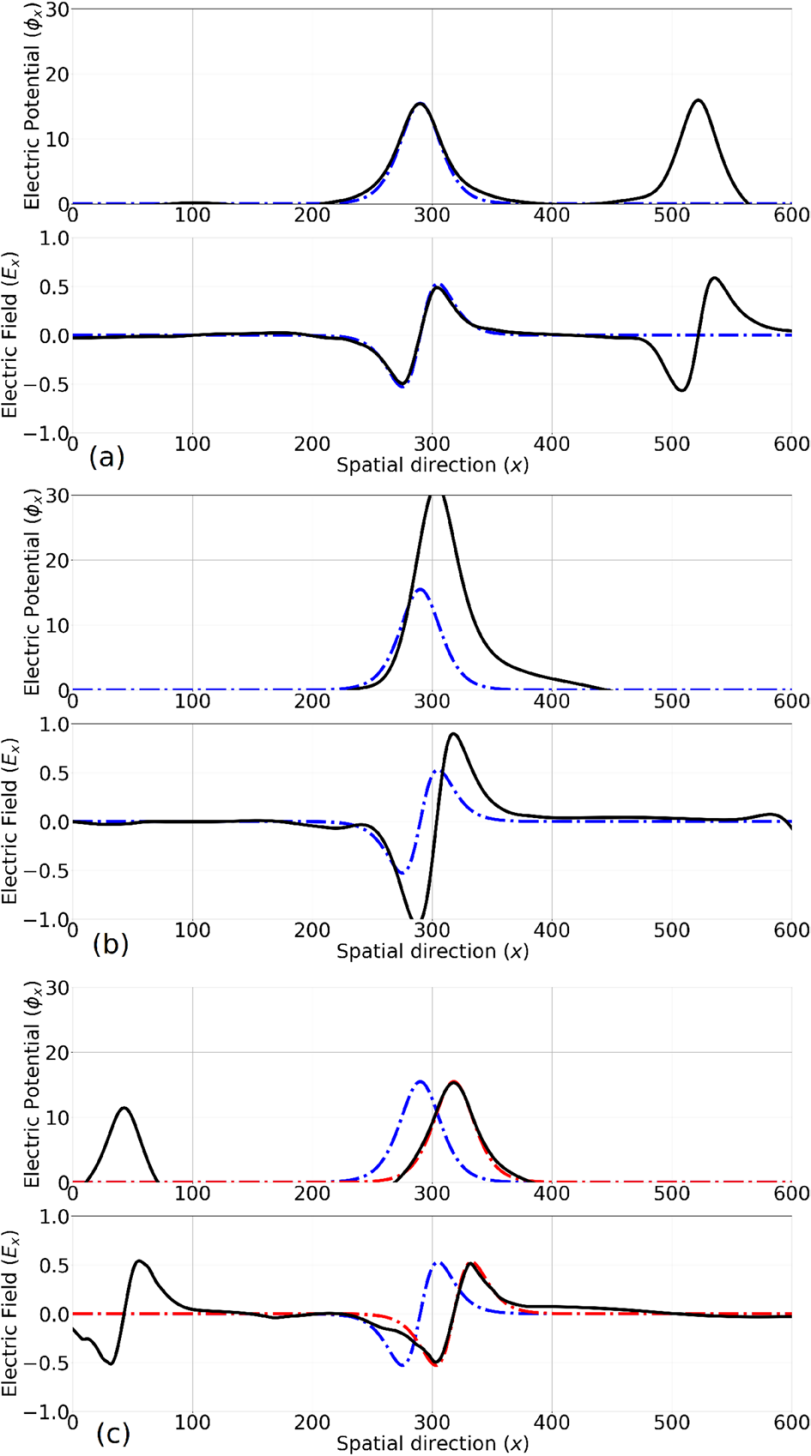
An overtaking collision between *EH*1 and *EH*3 is presented by plotting the electrostatic potential and the electric field profile in the co-moving frame of *EH*1 at three snapshots: (**a**) before ($\tau =2.35$), (**b**) during ($\tau =3.20$) and (**c**) after ($\tau =4.27$) the collision. The dotted curves show the fitted profile (${sech}^2$ before (blue) and after (red) the collision, for *EH*1. A shift in the position of the first EH can be witnessed (note the difference between the red and the blue curves) manifesting a phase shift, as intuitively expected.

Although the previous simulations demonstrate the stability of these EHs, the strongest test of the stability is their interaction via an overtaking collision when they overlap in the velocity direction. In an overtaking simulation, we have used two EHs e.g. *EH*1 and *EH*3. Figure 5 presents the temporal evolution of electric field/potential around the collision time $\tau =3.2$ in a frame moving with $M=45$. Both EHs survive the collision, and their respective velocity stays the same. Focusing on *EH*1, displacement can be witnessed after the collision. A phase shift can be measured by comparing EH profile with the red line, which is an extrapolation of an unperturbed path of this EH. This displacement is similar to the well-known effect of “phase shift” which observed to happen in mutual collisions of solitons^25–29^.

**Figure 6:**
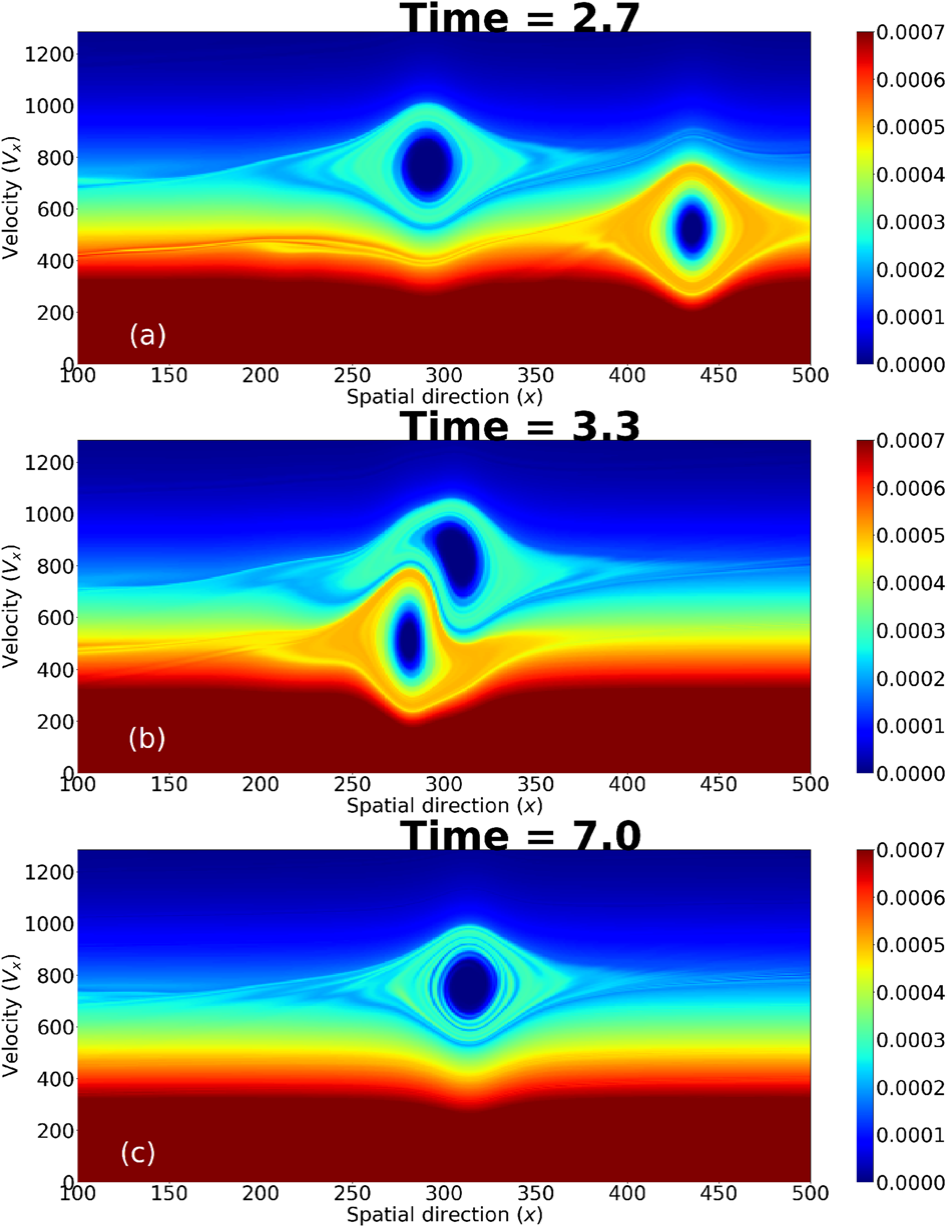
The electron phase space is presented for an overtaking collision between *EH*1 and *EH*3 in the co-moving frame of *EH*1. There is a substantial overlap in velocity direction (**a**). During collision, the interaction is strong (**b**). After the collision, *EH*1 reappears un-altered in (**c**).

We show in Fig. 6 the electron df during the overtaking collision, which demonstrates the considerable interaction between the EHs during the collision and their overlapping on velocity direction. Yet after the collision the *EH*1 is largely unperturbed, modulo the observed phase shift. Interestingly, data fitting has shown that the ${{\,\mathrm{sech}\,}}^2$ curve form approximates the numerical data better than any other exponent, including the (expected, arguably) ${{\,\mathrm{sech}\,}}^4$ form (see Eq. 39 in^14^).

## Discussion

In summary, we have provided a method to produce high-speed nonlinear solutions which move at a speed beyond the electron thermal speed. We showed that these electron holes are stable, retain their profile through collisions and remain so in the entire duration of the simulation. For mutual collisions with considerable overlap in the velocity direction, the EHs display a “phase shift” This phase shift represents a signature of soliton behavior and hence suggests that these EHs can be considered as solitons (at least approximately). This has been suggested for much lower-speed EHs before^5^ but without the observed “phase shift” reported here.

## Methods

### Equation set

The scaled Vlasov-Ampère system of equations forming the basis of our simulation reads:

1 $$\begin{aligned} \frac{\partial f_s(x,v,t)}{\partial t} + v \frac{\partial f_s(x,v,t)}{\partial x} + \Upsilon _s E(x,t) \frac{\partial f_s(x,v,t)}{\partial v}&= 0, \end{aligned}$$

2 $$\begin{aligned} \frac{\partial E(x,t)}{\partial t}&=\sum q_s J_s(x,t) \end{aligned}$$

where $s = i,e$ represents the corresponding species, i.e. ions and electrons respectively. The factor $\Upsilon _s$ takes the values $\Upsilon _e= -1836$ and $\Upsilon _i= 1$. The normalized charges are $q_e = -1$ and $q_i = 1$. The above equations are coupled by integrations for each species, viz. $J_s(x,t) = \int f_s(x,v,t) v dv$ in order to form a closed set of equations for *J*, denoting the current (contribution) generated by by species *s*. To derive the above (dimensionless) equations, all physical quantities were normalized to suitable scales related with ionic parameters, i.e. mass ($m_s$) was divided by the ion mass ($m_i$), temperature ($T_s$) by ion temperature ($T_i$), charge ($q_s$) by the elementary charge (*e*), time ($\tau$) by the ion plasma period ($\omega _{pi}^{1/2} = {\big (\frac{n_{i0} e^2}{m_i \epsilon _0}\big )^{-\frac{1}{2}} }$), and length (*L*) by the ion Debye length ($\lambda _{Di} = \sqrt{ \frac{\epsilon _0 K_B T_i}{n_{i0} e^2} }$). Here, $K_B$ is Boltzmann’s constant and $\epsilon _0$ is the permittivity of free space.

### Simulation code

We have employed the Gkeyll simulation framework^33^ to solve the Vlasov-Ampere system of equations^34–36^. Gkeyll discretizes the equations using the discontinuous Galerkin finite element method in space, with a strong stability-preserving Runge–Kutta method in time. We have adopted a piecewise cubic Serendipity Element space for the basis expansion^37^ (further details can be found in Refs.^34,36^). The Gkeyll method has been compared to the standard PIC method, where it was demonstrated that the effective phase space resolution of the method is very high, permitting detailed studies of df dynamics. Such high accuracy is of paramount importance for the resolution of EH dynamics in phase space^38^.

### Parameters

In our study, the temperature and mass ratio are $\frac{T_e}{T_i}=100$ and $\frac{m_i}{m_e}=1836$. The initial distribution function $f_0$ is considered to be the Maxwellian df ($=D_m$). The size (length) of the simulation box is $l=1000$ in the *x*-direction. In the *v* direction for each species, we have different limits: for the electrons we have $v=(-6, 6) v_{th_e} = (-2571,2571)$ and for the ions we have $v=(-10, 10)$, where $v_{th_e} = \sqrt{\frac{T_e}{T_i}\frac{m_i}{m_e}}\approx 428.5$ is the electron thermal velocity. The number of grid cells in each direction is $n_X = 2000$, $n_V = 1000$ for both electrons and ions. The time step $\,{\text {d}}{\tau }\approx 10^{-5}$ is chosen in order to fulfill Courant-Friedrichs-Lewy (CFL) condition^39,40^.

The electron hole speed ($v_{EH}$) is expressed by the “Mach number”, which is defined as the ratio $M = \frac{v_{EH}}{c_s}$, where $c_s = \sqrt{1+\frac{\gamma _e T_e+\gamma _i T_i}{m_i}}$ is the ion sound speed. Assuming $\gamma _e = \gamma _i = 3$ (heat capacity ratio), $T_e = 100 T_i$ and $m_i = 1$, the ion sound speed in our simulations is $c_s =\sqrt{304} \approx 17.43$.

### Iterative method to find stable solutions

Our method follows the BGK method and starts by adopting an arbitrary function for the electrostatic potential $\big (\phi (x)\big )$ and by choosing the value of the electron hole speed $(v_{EH})$. We then use the ELIN df to produce the electron distribution function. Given that the potential profile provides the charge density $(\rho (x))$, and using the Schamel df for the ions to obtain $n_i(x)$, we then use the total charge density (profile) $n_e(x) = \rho (x)-n_i(x)$ as a “guiding equation” for the ELIN df and thus construct the electron hole. We have adopted, to start with, the simplest form of potential profile suggested for electron holes i.e. $\phi = A {{\,\mathrm{sech}\,}}^p (x/L)$ in which $p=2$ and *A* and *L* are the EH amplitude and length, respectively. The amplitude and length (values) are chosen randomly; however the system will damp/break the forced profile if it is not close-enough to a self- consistent nonlinear solution. The resulting electron hole may have different size and velocity, but with an iterative process, one can find the combination of $\{A, L\}$ for which the solution will be stable enough for a specific (chosen) velocity value. Since we are not aware of the nonlinear dispersion relation, i.e. a relationship between $\{A, L, M, p\}$ for the exact nonlinear solution(s), a sequence of trials is performed to iterate to the correct combination of $\{A, L, p\}$ for a given *M*. In the simulations presented here three electron holes were studied, e.g.

- *EH*1: $$M= 45, A=19, L=22.5, p=2$$
- *EH*2: $$M=-40, A=9.5, L=22.5, p=2$$
- *EH*3: $$M= 30, A=19, L=22.5, p=2$$.

### Elin DF method to construct electron holes

In order to explain our novel distribution function approach, firstly we need to represent the Schamel distribution function in energy-dependent format. Here we briefly discuss this, more details can be found in the reference^19^. Schamel approach devides the distribution function into two parts, namely free and trapped particles which are separated by a separatrix.

Focusing on the free particles, the following steps are taken to determine their distribution function ($f_f$), assuming a pulse moving with a velocity ($v_{EH}$) in the laboratory frame:

1. the shifted kinetic energy is found in the co-moving frame: $\varepsilon '_{K_{sh}}=|\varepsilon _K'-\varepsilon _{\phi }|$ where $\varepsilon _{\phi }=q\phi$, $\varepsilon _K' = \frac{1}{2} \frac{m}{T} v'^2$ and $v'= v - v_{EH}$ is the velocity in the co-moving frame.
2. the shifted kinetic energy is calculated in the laboratory frame: $\varepsilon _{K_{sh}} = \frac{1}{2} \frac{m}{T} v_{sh}^2$ in which $v_{sh} = v'_{sh} + v_{EH}$ and subsequently $v'_{sh} = \text {sign} (v') \sqrt{2\varepsilon '_{K_{sh}}/m}$.

Free particles fulfill the condition $\varepsilon _K'>\varepsilon _{\phi }$. Note that, in order to calculate the df at point *v*, we use the df at the point $v_{sh}$, which can be written as $f=D_g(\varepsilon _{K_{sh}})$ in energy format. Here, $v_{sh}$ presents the velocity of particles before their interaction with the potential profile. By $D_g$ we denote a general distribution function satisfying the Vlasov equation, i.e. in principle any function depending on the constant(s) of motion. Here, the energy is used to construct a valid function. Well-known examples of $D_g$ are the Maxwell-Boltzmann df, the $\kappa$ df^41–44^ and the Cairns^45^ distribution function(s).

In other words we trace the characteristics of the particle back in phase space. Then we use the value of df at $v_{sh}$ as the value of df for *v* since the df stays constant on the characteristics of Vlasov equation^46^.

The distribution function of trapped particles ($f_t$) which are subject to the trapping condition ($\varepsilon _K'<\varepsilon _{\phi }$) can be achieved by following the steps below:

1. the shifted kinetic energy is found in the co-moving frame: $\varepsilon '_{K_{sh}}=|\varepsilon _K'-\varepsilon _{\phi }|$, using a Maxwellian df on top of this kinetic energy with a coefficient $\beta$, will provide the shape of trapped distribution function: $f_{shape} =D_m(\beta \varepsilon '_{K_{sh}})= \exp (-\beta \varepsilon '_{K_{sh}})$
2. In order to have continuity between trapped and free df where they meet in the velocity direction, $f_{shape}$ is multiplied by $f_{base} = D_g(\varepsilon _S)$. Hence $f_t = f_{base} \times f_{shape}$.

Here, $D_g(\varepsilon _S)$ stands for the distribution function at the separatrix where $\varepsilon _K'=\varepsilon _{\phi }$ and works as a constant value which can increase or decrese the $f_{t}$, in order to adjust it with the free distribution function. The second component, $f_{shape}$ is velocity-dependent and is controlled by $\beta$. It may appear in three qualitative shapes, i.e. *flat*, a *bump* or a *hollow* curve, if $\beta = 0$, $\beta > 0$ or $\beta < 0$, respectively (see Fig. 1).

Hence, the total form of the Schamel distribution function^12^ can be written in terms of the energy as: $f=a f(\varepsilon _K)$ in which *a* is a normalization constant and

3 $$\begin{aligned} f(\varepsilon _K) = \left\{ \begin{array}{lr} f_f = D_g(\varepsilon _{K_{sh}}) &{}\text {if} \ \varepsilon _K'>\varepsilon _{\phi }\\ D_g(\varepsilon _S) &{}\text {if} \ \varepsilon _K'=\varepsilon _{\phi }\\ f_t = D_g(\varepsilon _S) D_m\Big (\beta |\varepsilon _K'-\varepsilon _{\phi }| \Big ) &{}\text {if} \ \varepsilon _K'<\varepsilon _{\phi }\\ \end{array}\right. \end{aligned}$$

One can understand the Schamel df as carving up a given general distribution function ($D_g$) around a particular velocity (hole velocity) and inserting a Maxwellian df with arbitrary temperature inside the hole ($f_{t}$).

In the above representation of the Schamel df we used the analytical form of $\phi (x)$. However, one can equally use the discretizied form of $\phi (x)$ (by deviding it into a *n* intervals of $\Delta \phi$). Schamel df can then be retrieved by $n\rightarrow \infty$ ( $\Delta \phi \rightarrow 0$). In terms of simulation approach, these two methods are equal since even when using the analytical approach, one had to use discretization for $\phi (x)$ and there is limit on how small $\Delta \phi$ can get.

In other words, to generate distribution function ($f_f$ and $f_t$) for each interval, we only need update the value of $f_{base}$ in our approach and repeat the process. This results in multiple carvings, each based on the previous distribution function and it recursively progresses.

We assume $\phi = A {{\,\mathrm{sech}\,}}^2 (x/L)$ as crude approach (stablished by trial and error in the beginning), and then we dicretize the first half of $\phi (x)$ into *n* intervals in the following form ($\Delta \phi = \frac{A}{n}$):

$$\begin{aligned} \phi (x_1)&= \Delta \phi \\ \phi (x_i)&= \phi (x_{i-1}) + \Delta \phi \\ \phi (x_n = \frac{L}{2})&= A. \end{aligned}$$

The second half will be the same as the first half except for a simple inversion. Hence we just build the first half of df and the second half is just simple inverted copy of it.

In this approach $\beta$ can be changed for each interval, and this add a new degree of freedom to the Schamel df. We call this ELIN (rEcursiveLy extendable distribution for a trapped populatIoN) distribution function. The distribution function for each interval can be presented by the following equation. In which the $D_g$ (in Schamel df) is replaced by the distribution function $f_{i-1}$ of previous interval and each interval has its own $\beta _i$:

4 $$\begin{aligned} f( \phi _i) = \left\{ \begin{array}{lr} f_f = f_{i-1}(\varepsilon _{K_{sh}}) &{}\text {if} \ \varepsilon _K'> q \Delta \phi _i \\ f_{i-1}(\varepsilon _S) &{}\text {if} \ \varepsilon _K'= q \Delta \phi _i \\ f_t = f_{i-1}(\varepsilon _S) D_m(\beta _i(|\varepsilon _K'-\varepsilon _{\phi }|)) &{}\text {if} \ \varepsilon _K'< q \Delta \phi _i \\ \end{array}\right. \end{aligned}$$

in which $f_0$ is the initial unperturbed df (here assuming Maxwellian df, i.e. $f_0 = D_m$). $\beta _i$ can change arbitrarily in order for moments of df to fit a “guiding equation” (here, the equation for the electron density). To obtain a smooth distribution function in the *x* direction, one can increase *n* until the numerically-desired level of smoothness is achieved. An example of the ELIN df profile is presented at Fig. 1 which shows 10 successive (carving) iterations with $\beta$ approaching zero from below (negative side). Note that since $\beta$ originates from a continuous guiding equation, hence their successive values follow a pattern and are not randomly chosen.

To conclude, we have introduced a new method for constructing electron holes within a kinetic framework, which relies on a successive multi-step extension of the Schamel df (here represented in energy-dependent form), i.e. the ELIN df method. The ELIN df adopts a continuously varying value for $\beta$, in contrast to the Schamel df where $\beta$ is a constant. This extension provides an infinite number of parameters for the ELIN df, which enables it to construct an electron hole for any given bell-shaped potential profile. In our computational approach, the number of free parameters in the ELIN df is finite and equals the number of intervals (*n*). We have adopted an iterative method (inspired by Newton’s iterative scheme), built on top of the ELIN df method, to find the stable solutions. Starting from an initial guess, in each iteration of this method firstly we use the ELIN df to build an electron hole and then utilize the Vlasov-Poisson simulation method to follow the temporal evolution of the electron hole for a short time. We use the potential profile at the end of each iteration as an input for the next round of iteration. After a few iterations, the initial and final potential profiles are close enough for this to be considered as a stable configuration, for closure. Then, one can move on to longer-time numerical experiments, to investigate the long-time evolution of these localized structures and their behavior through mutual collisions. As a representative set, three stable solutions (i.e. EH1, EH2 and EH3; see above) have been reported in detail.
